# Referral patterns for retinoblastoma patients in Ethiopia

**DOI:** 10.1186/s12913-023-09137-9

**Published:** 2023-02-20

**Authors:** Sadik Taju Sherief, Fran Wu, Jacquelyn O’Banion, Tiliksew Teshome, Helen Dimaras

**Affiliations:** 1 Department of Ophthalmology, Menelik II Hospital, Addis Ababa, Ethiopia; 2 Department of Ophthalmology, School of Medicine, College of Health Sciences, Addis Ababa University, P.O. Box 9086, Addis Ababa, Ethiopia; 3 Department of Ophthalmology, Emory University, Atlanta, GA USA; 4 Department of Ophthalmology and Vision Science, The Hospital for Sick Children and University of Toronto, Toronto, Canada

**Keywords:** Retinoblastoma, Lag time, Socio economic factors, Sub Saharan African Countries, Public Health

## Abstract

**Background:**

Increased lag time between the onset of symptoms and treatment of retinoblastoma (RB) is one of the factors contributing to delay in diagnosis. The aim of this study was to understand the referral patterns and lag times for RB patients who were treated at Menelik II Hospital in Addis Ababa, Ethiopia.

**Method:**

A single-center, cross- sectional study was conducted in January 2018. All new patients with a confirmed RB diagnosis who had presented to Menelik II Hospital from May 2015 to May 2017 were eligible. A questionnaire developed by the research team was administered to the patient’s caregiver by phone.

**Results:**

Thirty-eight patients were included in the study and completed the phone survey. Twenty-nine patients (76.3%) delayed seeing a health care provider for ≥ 3 months from the onset of symptoms, with the most common reason being the belief that it was not a problem (96.5%), followed by 73% saying it was too expensive. The majority of patients (37/38, 97.4%) visited at least 1 additional health care facility prior to reaching a RB treatment facility. The mean overall lag time from noticing the first symptom to treatment was 14.31 (range 0.25–62.25) months.

**Conclusion:**

Lack of knowledge and cost are major barriers to patients first seeking care for RB symptoms. Cost and travel distance are major barriers to seeing referred providers and receiving definitive treatment. Delays in care may be alleviated by public education, early screening, and public assistance programs.

**Supplementary Information:**

The online version contains supplementary material available at 10.1186/s12913-023-09137-9.

## Background

Retinoblastoma (RB) is the most common intraocular malignancy of childhood, representing approximately 2.0% of all pediatric malignancies [1]. It is the most frequent intraocular malignancy of the eye in childhood occurring in early childhood; two-thirds are diagnosed before 2 years of age, and 95% before 5 years [2]. The incidence in various well-studied population groups around the world varies from 1 in 15,000 to 1 in 20,000 live births [3, 4], and is increasing in regions where the majority of patients survive and have affected children of their own [5].

The most common presentation of RB is leukocoria but if this early sign does not prompt the family to seek care, the tumor can continue to grow and spread relatively undetected until it may be too late for a cure, which can happen within months. In many low-income (LIC) and middle-income countries (MIC) presentation of RB is often late, and characterized by orbital involvement and metastasis [6–8]. This aggressive tumor grows quickly, metastasizes early, and can be fatal; however, it is also curable if diagnosed and treated early. In general, survival from retinoblastoma in LIC, lower MIC, upper MIC, and high-income countries (HIC) is 30%, 60%, 75%, and 95%, respectively [6]. Yet even within these national income categories, there is wide variation between treating RB facilities; for example, single institution studies from LICs and upper MICs report survival of up to 73% [9] and 96% [10], respectively.

Likewise, the burden of disease is much greater in LICs and MICs, where an estimated 84% of all children with cancer in the world reside [11]. Survival and visual outcome in RB are dependent on the severity of disease at time of presentation, so early detection is paramount to survival; however, children in East Africa present an average of 9 to 11 months later than their counterparts in the US and Canada, leading to poor survival rates [4].

Ethiopia has one of the highest estimated burdens of RB in sub-Saharan Africa [12]. RB in Ethiopia is characterized by delayed presentation and advanced disease [8, 13]. Menelik II Hospital is Ethiopia’s most advanced RB treatment center. This study was conducted to understand the path from first onset of symptoms to arrival at Menelik II Hospital in order to address delays in accessing timely care.

## Methods

### Study design and patient involvement

This single-center cross-sectional study was conducted as a part of a larger study entitled “Health Systems, Treatment, and Outcomes of RB in Ethiopia, and Mental Health of Primary Caregivers” with ethical approval from Institutional Review Board of Addis Ababa University (Protocol Number 101/17/Oph). Verbal informed consent was obtained from parents or guardians of the participants and procedure of Verbal informed consent was approved by the Review Board of Addis Ababa University. While patients were not involved as research partners in this work, we intend to disseminate the main results to participants. We will seek patient involvement in the development of an appropriate method of dissemination (e.g. plain language summary) and subsequent action to address the findings in practice.

### Study subjects

Caregivers (defined as parents or legal guardians) of RB patients who presented to Menelik II Hospital (Addis Ababa, Ethiopia) from May 2015 – May 2017 (inclusive) were eligible for this study. Caregivers of RB patients observed over the study period were included after (i) confirmation of true diagnosis of RB and (ii) identification of cross‑referred cases to avoid duplicate counting. In the case of more than one caregiver, the primary caregiver who knew the details regarding the patient and who brought the patient to Menelik II Hospital was selected.

### Data collection

A structured survey was designed to record patient experiences prior to being seen at Menelik II Hospital. The survey was administered by phone in January 2018, in the Amharic or Oromifa languages and took 30–45 min to complete.

An English version of the survey is provided in Additional file 1. Data points included: age of patient, sex, home address, laterality, family history, presenting signs and symptoms, health facility/site of presentation, health facility/site referred to, time from first noticing signs of RB to clinical diagnosis, time from clinical diagnosis to presentation at a Menelik II Hospital. To determine the referral pattern of patients from the onset symptoms to the time they were seen at Menelik II Hospital, the medical records of the included patients were examined.

We adapted the definition of lag time from a previously published study in the UK [14], making chages to account for the health care and referral systems in Ethiopia. Namely, overall lag time was defined as the interval between the date of first noticing the symptom by a caregiver to the date of treatment. We divided the overall lag time into 3 segments: lag 1 referred to the time from the recognition of symptoms to the initial consultation with first care provider (delay in the initial visit), lag 2 referred to the time from the initial consultation to the diagnosis of RB (delay in diagnosis), and lag 3 referred to the time from diagnosis to treatment (delay in treatment). Thus, the lag time is not necessarily correlated to the referral pathway.

### Data analysis

Basic descriptive statistical analysis was done using IBM SPSS Statistics (Version 21.0) software.

## Results

### Study subjects

**Fig. 1:**
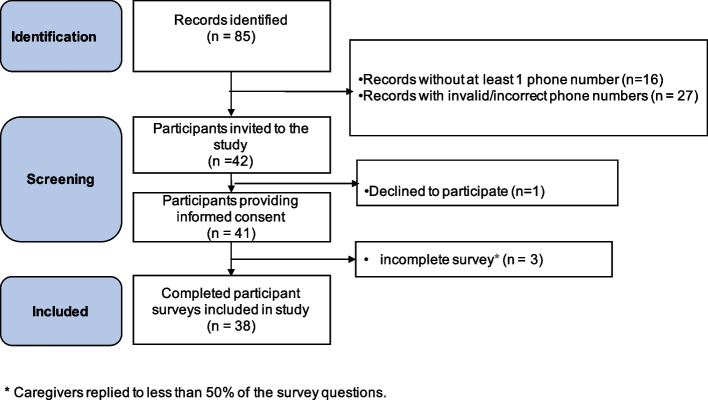
Study Participant Selection. Of 85 eligible records, 42 had valid phone numbers and were invited to participate. All provided informed consent, however 4 were excluded due to incomplete surveys (i.e. caregivers replied to < 50% of the survey questions). The final study number was 38

There were 85 new RB patients seen at Menelik II Hospital who presented from May 2015-May 2017. Out of the 85 patient records, 16 were excluded for not having a phone number listed. Of the 69 remaining, 27 were excluded due to invalid/incorrect phone numbers, leaving 42 caregivers who were invited to participate in the study. One declined, resulting in a, a total of 41 subjects enrolling in the study. However, 3 study participants were excluded from the analysis because they completed less than 50% of the survey questions, for a final study number of *n* = 38 (Fig. 1).

### Study subject demographics

Participants included 25 males and 13 females, for a male to female ratio of 1.9:1. Considering the relationship of the study participants to the patients, 22 were fathers (57.9%). Most participants came from the Oromia region (39.5%) and none were from Harar, Dire Dawa, Afar or Benishangul-Gumuz.

### Patient demographic and clinical characteristics

The mean age at noticing the first sign of RB was 13.92 ± 17.16 months (range 0–84 months) and the mean age at presentation to the first care provider was 22.38 ± 16.95 months (range 3–84 months). At time of the study, the caregivers reported that 20/38 (52.6%) patients in their care were alive and 18/38 (47.4%) had passed away from advanced disease (Table 1).

**Table 1:** Socio-demographic characteristics of patients with retinoblastoma

Patient Characteristics	mean ± SD (median, range)	n	%
**Laterality**			
Unilateral		27	71.1
Bilateral		11	28.9
**Sex**			
Male		25	65.8
Female		13	34.2
**Status at follow-up**			
Alive		20	52.6
Dead		18	47.4
**Age at onset ( months)**			
All cases (*n* = 38)	13.92 ± 17.16 (8, 0–84)		
Bilateral	6.73 ± 6.59 (5, 0–21)		
Unilateral	16.85 ± 19.28 (11, 0–84)		
**Age at Presentation at first care provider(months)**			
All	22.38 + 16.95 (24, 3–84)		
**Age at diagnosis (months)**			
All cases (*n* = 38)	27.62 ± 17.22 (25, 3–86)		
Bilateral	17.33 ± 7.51(15, 6–36)		
Unilateral	30.93 ± 17.37(29.5, 3–86)		
**Distance (km) home to…**			
**First Care Provider (*n* = 38)**	37.08 ± 85.83 (11, 1–450)		
< 100 km		34	89.5
100-200 km		2	5.3
> 200 km		2	5.3
**Second Care provider (*n* = 37)**	257.53 ± 253.99(197.5, 10–866)		
**RB treatment Center (*n* = 38)**	386.02 ± 85.83 (332, 20–1125)		
< 100 km		9	23.7
100- 300 km		8	21.1
301-500 km		9	23.7
> 500 km		12	31.6
**Cost (Birr) of …**			
travel from home to first care provider	42.8 ± 99.64 (10, 0–560)		
travel from home to RB treatment center	3446.4 ± 19,981.72(15, 0–120,000)		
care at first care provider	233 ± 370.8 (100, 5–1550)		
care at RB treatment center	7350.6 ± 39,906.4 (80, 0–240,000)		
	**Mean (median, range)**		
**Number of care providers visited before arriving at the RB treatment center**	1.5 (2, 0–2)		

### Referral pathway

**Fig. 2:**
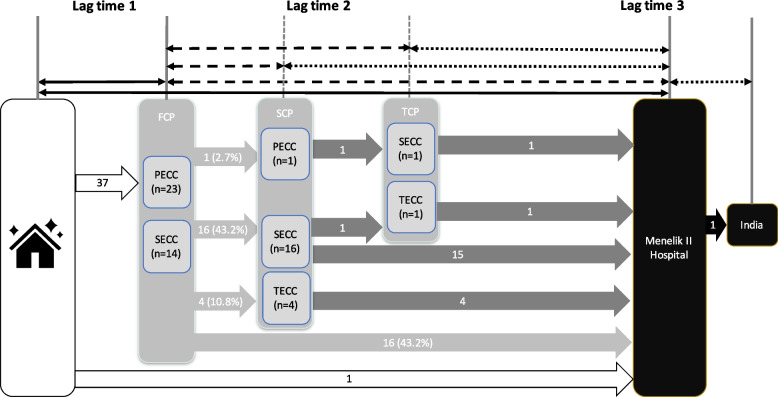
Referral Pathway from home to Menelik II Hospital. Of 38 patients, 1 self-referred directly to Menelik II Hospital, while 37 visited at least 1 additional health facility prior to arriving at Menelik II Hospital. One patient refused care at Menelik II Hospital and self-referred to a center in India. FCP = First Care Provider; SCP = Second Care Provider; TCP = Third Care Provider; PECC = Primary Eye Care Center; SECC = Secondary Eye Care Center; TECC = Tertiary Eye Care Center

The majority of patients (37/38, 97.4%) visited at least 1 additional health care facility prior to reaching a RB treatment facility, while 1 self-referred directly to Menelik II Hospital (Fig. 2). Fourteen patients (36.8%) visited 1 and 29 patients (76.3%) visited 2 facilities between home to Menelik II Hospital (Table 2). On average, RB patients visited 1.5 care providers prior to arriving the final RB center (Table 1). The diagnosis of RB was made at Menelik II Hospital in 21 (55.3%) of the patients (Table 2).

**Table 2:** Referral pathway and location of diagnosis by lag time in retinoblastoma diagnosis

**Patient Characteristics**	**n**	**%**	Mean ± SD (median, range) overall lag time (months)	Mean ± SD (median, range) Lag Time 1 (months)	Mean ± SD (median, range) Lag Time 2 (months)	Mean ± SD (median, range) Lag Time 3 (weeks)
			*n* = 38	*n* = 38	*n* = 38	*n* = 38
**Patients**	38		14.31 ± 9.3 (9.3, 0.25–62.25)	7.97 ± 8.98 (4, 0–36)	5.2 ± 9.3 (1, 0–38)	3.18 ± 8.82 (1, 0–52)
**Referral Pathway**						
Home to Menelik II	1	2.7	4.25	4	0	1
Home—1 Facility—Menelik II	14	36.8	12.46 ± 10.99 (12.63, 0.25–37)	7.5 ± 6.94 (8, 0–22)	3.57 ± 6.52 (1, 0–24)	4.71 ± 13.62 (1, 0–52)
Home—2 Facilities—Menelik II	22	59.5	15.37 ± 16.04 (9.25, 0.25–62.25)	8.5 ± 10.37 (3.5, 0–36)	5.68 ± 10.54 (1, 0 = 38)	1.73 ± 3.22 (1, 0–16)
**Timing & Location of Diagnosis**						
1st Facility	3	7.9	5.92 ± 6.41 (4,25, 0.5–13)	1.33 ± 2.31 (0, 0–4)	0	18.33 ± 29.16 (2, 1–52)
Menelik II	1	2.6	4.25	4	0	1
Other	2	5.3	6.75 ± 8.84 (6.75, 0.5–13)	0	0	27 ± 35.36 (27, 2–52)
2^nd^ Facility	29	76.3	14.12 ± 14.31 (14.1, 0.25–62.25)	7.3 ± 7.01 (5, 0–24)	6.28 ± 10.35 (2, 0–38)	2.1 ± 3.99 (1, 0–16)
Menelik II	14	36.8	13.54 ± 11.75 (13.25,0.25–37)	7.93 ± 6.99 (8, 0–22)	5.07 ± 7.9 (1.5,0–24)	2.14 ± 4.04 (1, 0–16)
Other	15	39.5	14.5		7.4	1.6
3rd Facility (all Menelik II)	6	15.8	17.04 ± 15.63 (15.3,0–37.25)	14.5 ± 15.46 (13.5,0–36)	2.3 ± 2.88 (1,0–6)	0.8 ± 0.41 (1, 0–1)
**Location of Diagnosis**						
Menelik II	21	55.3	14.09 ± 12.55 (12.3, 0–37.25)	9.62 ± 10.12 (6, 0–36)	4.04 ± 6.72 (1,0–24)	1.71 ± 3.32 (1, 0–16)
Other	17	44.7	13.74 ± 16.05 (9.25, 0.25–62.25)	5.94 ± 7.11 (3, 0–24)	6.53 ± 11.84 (1, 0–38)	5 ± 12.64 (1, 0–52)

### First care provider – lag time 1

The most common first symptom of disease, leading parents to seek care at the first care provider level, was leukocoria (37/38, 97.4%), followed by strabismus (1/38, 6.2%). The mean age when a caregiver noticed an eye symptom for the first time was 13.92 (range 0–84) months. Bilaterally affected patients were younger at the onset of symptoms, with a mean age of 6.73 (range 0–21) months compared to 16.85 months in unilateral cases (range 0–84). On average RB patients travelled 37.08 ± 85.83 km (range 1–450 km) from their home to the first care provider and paid 233 ± 370.8 Birr (range 5–1550 Birr) for the care (Table 1).

Twenty-three (60.5%) RB patients were seen in health facilities where RB care was not provided as their first contact point. Two patients (5.3%) went to traditional healers as a first-line treatment. Twenty-five (65.8%) patients were first seen at community health centers or hospitals without ophthalmologists (Table 3). For 20/38 (52.6%) participants, the top reasons for choosing their first care provider included proximity to home and recommendation by health extension workers (HEW) or friends (Table 3).

**Table 3:** Health Care providers by the types of professionals, reasons for choosing and delay for seeking care

**Characteristics**	**Number (n)**	**Percentage (%)**
**First Care Provider** ***n* = 38**		
Health Facility		
Primary eye care center	23	60.5
Secondary eye care center	14	36.9
Menelik II	1	2.6
Care Provider by profession		
Ophthalmologist	11	28.9
General Practitioners	6	15.8
Nurse	14	36.8
HEW	5	13.2
Traditional Healers	2	5.3
Reasons to choose the FCP		
I had no choice	10	26.3
I trust the person /center	8	21.1
Referred by friend	8	21.1
Referred by HEW	5	13.2
Refereed by friend and I trust the center	6	15.8
Refereed by friend and I had no choice	1	2.6
Reasons for Delay > 3 months to seek care after noticing symptom *n* = 29		
Didn’t think it was a problem and too expensive	11	37.9
Didn’t think it was a problem, too far and too expensive	9	31
Didn’t think it was a problem	6	20.7
Didn’t think it was a problem and too far	2	6.9
Too expensive	1	3.5
**Second Care Provider** ***n* = 37**		
Health Facility		
Primary eye care center	1	2.7
Secondary eye care center	17	45.9
Tertiary eye care center	4	10.8
Menelik II	15	40.5
Care Provider		
Pediatrics Ophthalmologist	17	45.9
General Ophthalmologist	16	43.2
General Practitioners	4	10.8

The mean lag time 1 at first facility was 1.33 ± 2.31 months (range 0–4 months). The mean lag time 1 increased to 7.3 ± 7.01 months (range 0–24 months) for patients who visited 2 health facilities prior to reaching Menelik II Hospital (Table 2). Most of the patients (29/38, 76.3%) were delayed seeing the first care provider for ≥ 3 months from symptom onset (Table 3). Even after reaching the first care provider facility, for 24/38 patients (63.2%) it took ≥ 1 week to be seen by the primary health care team.

The leading cause of delay in seeking care was “Did not think there was a problem”, reported by 28/29 (96.5%) of the patients who presented ≥ 3 months after noticing the symptom (Table 3). Patients were referred to secondary eye care facilities where RB services were not provided during the study period by 16/36 (44.7%) of the first-care providers.

### Second care provider

Thirty-seven participants sought care from a second care provider (Table 1). Though it was their second contact point for seeking care, 21/37 (56.8%) patients visited health care facilities which did not provide RB diagnosis and treatment. Out these, 17/21 (81%) participants were seen at facilities with an ophthalmologist on staff (Table 3). The mean distance from home to the second care provider was 257.53 ± 253.99 km (range 10–866 km) (Table 1). Despite the fact that four of the second care providers were designated as tertiary eye care centers, they did not provide RB services during the study period.

### Diagnosis – lag time 2

The mean lag time 2 was 5.2 + 9.3 months (range 0–38 months). The mean lag time 2 was prolonged in those patients who visited 2 facilities prior to Menelik II Hospital (6.28 months) (Table 2). and seen other than Menelik II hospital as their second visit (7.4 months) (Table 2).

### RB treatment center – lag time 3

All of the study participants (38/38) were seen at Menelik II Hospital (RB Treatment Center) on their first, second, third or fourth encounter (Fig. 2). Four patients (10.5%) were self-referred. One patient refused care at Menelik II Hospital and self-referred to a center in India (Fig. 2).

The mean distance from home to RB treatment center was 386.03 ± 85.83 km (range 20–1125 km) (Table 1). The average costs for travel (from home to Menelik II Hospital) and treatment was 3,446.4 ± 19,981.72 and 7,350.6 ± 39,906.4 Ethiopian Birr respectively (Table 1).

The mean lag time 3 was 3.18 ± 8.82 (range 0–52) weeks. Patients who visited 2 facilities before Menelik II Hospital had a smaller lag time 3 (1.73 weeks) than those who visited just 1 facility (4.71 weeks) (Table 2).

### Overall lag time

The mean overall lag time from noticing the first symptom to the treatment of RB at the RB center was 14.31 ± 9.3 (range 0.25–62.25) months (Table 2). Fifteen children (15/38, 39.5%) waited a month or more to get treatment once the diagnosis was settled. For patients where the diagnosis of RB was made at Menelik II Hospital as the third health facility, the overall lag time was 17.04 months (Table 2). Strikingly, we observed 18/38 children (47%) with an overall lag time of 12 months or more (Table 4). There was a statistically significant association of overall lag time > 12 months with mortality (*P* = 0.0496 Table 4). Treatment was refused by 2/38 patients (Table 4). For the 36 patient who were treated, therapies were variable and often multimodal, and included enucleation, chemotherapy, laser therapy, and radiation (Table 4). When looking at survival and mortality, a significant difference was observed between groups treated with and without enucleation (*P* = 0.0113, Table 4). Those patients without enucleation were advanced RB cases treated with chemotherapy with or without radiation or laser, with a high risk of mortality from distant metastasis.

**Table 4:** Survival of patients with retinoblastoma by overall lag time and treatment type

	**All Patients**		**Status at last follow-up**				***P*-value**
			Alive		Dead		
	n	%	n	%	n	%	
**All Patients**	38	100%	20	53%	18	47%	-
**Overall Lag time**							
< 12 months	20	53%	14	37%	6	16%	0.0496*
≥ 12 months	18	47%	6	16%	12	32%	
**Treatment Adherence**							
Adhered to Treatment	36	95%	20	53%	16	42%	0.2176
Treatment Refusal	2	5%	0	0%	2	5%	
**Treatment Type (*n* = 36)**							
Enucleation	26	72%	18	50%	8	22%	0.0113*
Enucleation only	8	22%	8	22%	0	0%	
Enucleation + chemotherapy	11	31%	8	22%	3	8%	
Enucleation + chemotherapy + laser	2	6%	2	6%	0	0%	
Enucleation + chemotherapy + radiation	5	14%	0	0%	5	14%	
No enucleation	10	28%	2	6%	8	22%	
Chemotherapy alone	7	19%	0	0%	7	19%	
Chemotherapy + radiation	1	3%	0	0%	1	3%	
Chemotherpay + laser	2	6%	2	6%	0	0%	

^*^Statistically significant *p*-value < 0.05

## Discussion

This is the first study in relation to the referral pattern of RB patients in Ethiopia, examining timing of each step in seeking care. This study assessed the experience of patients from first noting signs of RB in their home, to arriving for treatment at Menelik II Hospital, Ethiopia’s most comprehensive RB treatment center. The study revealed the magnitude of lag time, specifically in the delay in treatment, as well as the relationship of caregiver knowledge, cost and travel distance to these delays. These findings have implications for developing health education campaigns and public awareness programs, and development of health care resources and counselling guidance for patient caregivers.

A prospective study from 11 RB centers showed that the lag time between first sign and treatment of RB was 5.4 times higher in LIC, 3.2 times higher in lower MIC and 1.6 times higher in upper MIC when compared to HIC [15]. The overall mean lag time observed in our study (14.31 months) was much longer than studied from UK (38 days) [14], North China (1 month) [16], Thailand (5 weeks) [17], Central and Southern China (54 days) [18], India (3 months) [19], Brazil (5.8 months) [20], and other sub-Saharan African countries like Kenya (6.8 months) [21] and Tanzania (10.6 months) [22], but shorter than a study from Mali (50 months) [23].

In comparison to other studies, the mean lag time 1 in our study population (7.97 months) was much longer than a report from China (8 days) [16] and UK (28 days) [14]. Participants in our study indicated that lack of knowledge was a reason for their late presentation, underscoring the importance of raising public awareness of the early signs of RB. This will have positive effects for pediatric eye conditions beyond RB, as leukocoria can also be indicative of Coats disease, persistent fetal vasculature, or familial exudative vitreoretinopathy, among others [24–26].

Lag time 2, the delay from first presentation to diagnosis by the first care providers, was more prolonged in our study population (5.2 months) than in UK (2 days) [14] and China (3 days) [16]. A lag time of this magnitude even though patients have already presented to a health facilities possibility indicates poor awareness of RB among health care providers or potentially incorrect choice of initial health facility by patient caregivers. The wide range of lag time 2 (0‑38 months) we observed could be due to the difference in diagnosis capacity of the primary care providers for patients with RB.

Lag time 3 represented the time to treatment of RB once the diagnosis was made. Though its much shorter than lag time 1 and 2 of our study population, lag time 3 in our study participants (3.18 weeks) is longer than that reported in UK (6 days) [14]. Besides, there is wide range in delay of treatment initiation for RB patients (0–13 months). The reasons for the delay in treatment could be multiple, but lack of standard operating procedure and a clear guideline at Menelik II Hospital can be reasons.

Prolonged lag time before initiation of RB treatment can lead to advanced disease presentation and poor patient outcomes. A study of 4351 RB patients from 153 countries from different national income status revealed that patients from LIC had a larger proportion of patients with symptoms of advanced disease compared to patients from HIC [27]. A Brazilian study showed that a six-month delay in diagnosis was linked to an increased risk of extraocular disease [28]. Similarly, a study from India revealed that prolonged duration of symptoms > 6 months was predictive of high-risk histopathologic features of RB [29]. In our study, prolonged lag time of 12 months or more was significantly associated with death (Table 4), suggesting that efforts to reduce lag time are imperative to save lives from retinoblastoma.

The literature points to several interventions which may be successful in reducing lag time. With respect to lag time 1, in Honduras a relatively inexpensive awareness program in conjunction with a national vaccination campaign was shown effective in an early diagnosis of retinoblastoma; they observed an increase in the number of referrals and, more importantly, a significant decrease (from 73 to 35%) in the occurrence of extraocular disease [30]. In the 1990s, in Brazil, an education program comprising primarily magazines and news articles about RB leading to earlier diagnosis and a reduced (from 56 to 17%) frequency of extraocular disease [31].

Interventions at the level of the health system may also prove effective to improve the referral pathway and facilitate connection of families to appropriate care; this would have been useful for the 21 patients in our study who were not referred to Menelik II Hospital after presenting at a primary or secondary eye care centre. In the Ethiopian health care delivery system, health extension workers are the main grassroots health work forces for promotive, preventive and basic curative services focused on common diseases, and other community-based health care services [32]. However, a previous study revealed that their knowledge regarding common eye diseases is limited [33]. Enhancing the health extension training program to include instruction on common signs of eye cancer in children may improve awareness and facilitate timely and appropriate referral of children with RB. Additionally, the delay in presentation to health facilities and diagnoses may be alleviated by public education through mass media and further training of primary and secondary health workers. Integration of screening for RB and other common pediatric eye diseases at vaccination or under five clinics may be another way to improve early detection of the cancer within the heath system.

In Ethiopia ophthalmologists work at secondary or tertiary health centers, but in this study we observed that the majority of the patients were first seen at primary health facilities, increasing the lag time to achieve diagnosis, and exposing patients to unnecessary costs. We also noted that the secondary health centers in which patients presented to prior to arriving at Menelik II Hospital did not provide RB treatment, though theoretically they should be able to provide some aspects of care; this requires further study. The underrepresentation of patients and caregivers from the regions of Harar, Dire Dawa, Afar or Benishangul-Gumuz may be reflective of affected patients ending up at health facilities other than Menelik II Hospital. However, it is also possible that low capacity for eye care in Afar and Bunshangul-Gumuz regions may be missing RB patients in those regions, warranting further study.

### Limitations

The study was limited as it relied on participant recall and might have resulted in response bias. Furthermore, missing contact information in the medical charts resulted in a small study participant number. Finally, there was a selection bias for patients who did eventually made it to the tertiary care center for treatment, omitting study of patients who were lost along the referral pathway or never made it to a point a first contact.

## Conclusions

Our results show that the RB patients in Ethiopia have long overall lag time from notice of first notice of sign to receiving treatment. Lack of RB awareness among caretakers, high cost and travel burden were significant barriers to receiving timely care. Delays in seeking care, diagnosis and on time initiation of treatment for patients with RB may be alleviated by public education and targeted capacity building in the Ethiopian health care system.

## Supplementary Information

**Additional file 1.**

## Data Availability

The datasets used and/or analysed during the current study available from the corresponding author on reasonable request.

## Abbreviations

LIC: Low income countries
MIC: Middle income countries
HIC: High income countries
PHCF: Primary Health Care Facility
RB: Retinoblastoma
