# Supplementary material for: Referral patterns for retinoblastoma patients in Ethiopia
Source: BMC Health Serv Res. 2023 Feb 20;23:172. doi: 10.1186/s12913-023-09137-9 (PMC9942339; doi:10.1186/s12913-023-09137-9)
Supplement: Supplementary file 1 — Additional file 1. [file 12913_2023_9137_MOESM1_ESM.doc]

# Additional file 1

**Referral Pattern of Retinoblastoma in Ethiopia**

**Data collection form**

This questionnaire is prepared for the purpose of conducting a study to assess the pattern of ocular trauma patients at department of ophthalmology, Menelik II referral hospital in Addis Ababa. In view of this you are kindly requested to fill out the questionnaires since your response is necessary in reflecting the reality

The data obtained from the questioner will only be used for research purpose.

1. General Information
2. Name of the patient ________________________________
3. Study ID/MRN _____________________________________
4. DOB ____________________________
5. Phone #1 _______________________________
6. Phone # 2_______________________________
7. Date of survey /call________________________
8. Result of call A. Valid B. Invalid
9. Interview
10. Person completing the survey ____________________________
11. Language ____________________________
12. Unable to complete survey due to language barrier? A. Yes B. No
13. Parent spoken to (Mother/Father /Other )
14. If Other , list relationship __________________
15. Name of caretaker __________________________________
16. Address (Region/Woreda/Kebele)________________________________________________
17. When did you first notice eye symptoms? Age of patient (months)/ Date (month/year) ______________
18. When did you first seek help? Age of patient/ Date (month/year)_______________________
19. If >3 month gap, why did you wait to see someone? Other (describe) ________________________________________________
20. Who did you go to when you sought help?
21. Name of provider
22. Name of clinic/hospital
23. Location of provider (Region/Woreda/Kebele)___________________
24. How far is the provider from your home? (km)______________________
25. Why did you see this provider? (check all that apply)

(describe)_________________________________

1. How much was travel, not including lodging (Birr)?
2. How much did it cost to see this provider (Birr)?
3. How long did it take from the time you tried to be seen to the time you were actually seen in person (weeks)?_______________
4. If you were not seen within a few days, what caused the delay?

(describe)_________________________________

1. What was the result of that visit (check all that apply)
2. Treatment/diagnostics (elaborate in another column)
3. Eye drops (describe)
4. Chemotherapy-how many weeks of therapy?
5. Laser (right, left, or both?)
6. Cryotherapy (right, left, or both?)
7. Enucleation (right, left, or both?)
8. Exenteration (right, left, or both?)
9. Radiation
10. Imaging CT/MRI
11. Lumbar puncture
12. Other (describe)_________________________________
13. If "(c) Referred" was chosen in previous questions
14. Name of clinic/hospital
15. Region
16. Woreda
17. Kebele
18. Distance from home (km)_____________________

K. Did you undergo the recommended tx or referral?

i. Yes, referral

Yes, tx complete If Yes when completed? Age of pt (months)____ month/year_____

iii. Yes, tx ongoing

iv. No why? ___________________

L. Is your child living?

i. Yes, please include me

ii. Yes, but don't include me

iii. No If not living, when did he/she pass away? Age (months)_____

Date (month/year) ___________
